# Differentiation of Remedies

**Published:** 1885-05

**Authors:** P. P. Wells

**Affiliations:** Brooklyn, N. Y.


### HOMŒOPATHIC PHYSICIAN,

A MONTHLY JOURNAL OF MEDICAL SCIENCE.

"If our school ever gives up the strict inductive method of Hahnemann, we are lost, and deserve only to be mentioned as a caricature in the history of medicine."—CONSTANTINE HERING.

Vol. V. MAY, 1885. No. 5.

### DIFFERENTIATION OF REMEDIES.

P. P. WELLS, M. D., BROOKLYN, N. Y.

The greatest perfection in specific prescribing is attained by him who has most carefully studied the differences in the actions of different drugs and acquired the power and the habit of their ready apprehension in his efforts to find the most similar remedy in the discharge of his clinical duties. The discovery and giving of this is *specific* prescribing. The greatest difficulty the beginner meets in his early attempts at this discovery, after that of getting his totality of the symptoms, is in the fact that he has found so many remedies in his search which seem to him, each, so just like the others, that before the group he is only confused, and it is difficult for him to see why any one of the group is not just as appropriate to his case as either of the others. All seem to him equally alike. The first step for such an one in making his selection is to dismiss from his thoughts all these similar elements of the actions of the members of this group. These are no aids in the discriminations his right selection of his curative requires. It is the similar in the action of the drug to the sick phenomena which he is to find. But as he finds symptoms more or less similar to these in several different drugs, it is evident his choice must be determined by those elements of the drug-action in which are differences; so that the problem of specific prescribing is for the most part made up of this twofold combination of similarity and difference. It is well to study drugs in this their twofold relation to their clinical use till it becomes the life-habit of our efforts as prescribers. In right discriminations of these is to be found the greatest possible successes of all true healers.

It is our purpose to make a few studies of the differentiation of remedies, such as every truly "scientific" (we like this much-abused but good word)—*i. e.*, homœopathic—prescription requires. We will first take up the three medicines which oftener than others present the difficulty of a common similarity to elements of acute diseases most frequently met in practice, viz.: *Acon.*, *Bell.*, and *Bry.* It is not seldom that even the most practiced and careful prescriber is at a loss for his choice of the right one for his case, which presents elements the like of which is found in the symptomatology of each of these drugs. It is not a matter of indifference which of them he selects. One of them may be his curative; all three cannot be. How shall he find the true one? Clearly by the guidance of other symptoms than these similar ones. The symptoms of the sick with which we have to do are all those aberrations of functions of disposition, mind, and body from the natural balance we call health. Then, if the question of selection is between these three, and we hesitate because of their seemingly equal similarity to the generalities of the case, we may examine as to the *disposition* of the patient, and the result is not seldom decisive of the choice. This is a most important department of symptomatology—and never to be neglected. The three remedies we have named are here found to be quite as different as they are similar in some of the more general phenomena of sick and drug action. The *disposition* of *Acon.* is *complaining*, and *loud* in its complaints and outcries. It believes *death near* and is *afraid* of it. These two features of the disposition of Acon. are very characteristic of the drug, especially the first. Cases may be met which require Acon. when there is not this fear of immediate death; but where it is present, and the other symptoms are such as to cause doubt as to which of the three named drugs shall be selected, it is decisive of the choice for Acon. But if this fear be met in cases where the symptoms cause doubt whether this drug or Ars. or Lach. or some other drug shall be given, then it falls into the category of the similar drug symptoms, and the choice of either for a curative is determined by other and, in this case, more specific symptoms. The choice is determined by the differences in these. But as to the first—the loud complaining—it is more of a decisive element as to the choice, and so universally is it present in cases which require this drug for their cure that the rule may be quite safely adopted to discard Acon. in all cases characterized by a quiet *morale.*

Bell., on the contrary, is generally characterized by *violence.* In whatever function of body or mind the drug develops its effects, these are likely to be marked by violence. The disposition, if it causes melancholy, may impel to suicide, or to silence and unwillingness to speak. The patient is easily made angry and is not pleased with anything, and if this is carried to the extent of delirium the patient strikes, bites, and is quarrelsome. Or the disposition may be subject to sudden alternations of opposite extremes [see Ign.], as from loquacity to silence, or from apathy to extreme sensitiveness, or from a joyous to a raging delirium, and the like.

Bry. is more characterized by depression than exaltation. Is *discouraged, timid, dreads the future;* is indisposed to thought; is *irritable, angry, quarrelsome;* desires absent things, which are rejected when brought; is given to *weeping.* So it will be seen the three remedies are easily differentiated by this element of their pathogenesis.

Then, as to the aspect the patient presents. This is likely to be the first, or one of the first, elements of the case to engage the prescriber's attention. If it be suggestive of Acon. he finds the face *red, hot,* perhaps *swollen, sweat* on the *forehead, upper lip,* and on the *cheek on which* the patient *has been lying;* and if the perspiration *be hot* it is to have weight in the decision of the choice of the remedy, and the more if there be perspiration over the general surface which is *hot.* This is so characteristic of Acon. that there should be important reasons in the case to justify its rejection and the choice of another remedy instead of this; and the more, if, with this peculiarity of the perspiration, there be with it the characteristic *morale* of the drug.

Bell. has also the *red, hot, turgid face,* or *sensation of burning* without redness, or a *hot forehead* with *cold cheeks,* or *hot face and head* with *cold hands and feet.* The eyes affected by this drug have a peculiar appearance—when once seen not easily forgotten. And when the like of this is met in a case of sickness, it is often properly decisive of its choice for the cure. The eyes are *prominent, red, injected, suffused, sparkling.* The redness is as if it were slightly obscured by the conjunctiva, as if this tissue were less affected than those beneath it. This peculiar expression of the eyes, where the other symptoms are like those of the drug, will often warrant its choice.

With Bry. the face is *pale, yellow,* or of an *earthy color,* or there may be *heat* of face with *redness* and *burning,* or circumscribed *red spots*, especially on the *zygoma* and *throat*. These appearances of the face, in the absence of the concomitants of Acon. and Bell., may be accepted as warrants for the selection of Bry.

The pains of Acon. are severe, and are, for the most part, intolerant of *touch* or *movement*. There is great soreness of the general surface. The *shooting* pains of Acon. are more *internal* than external; and the analogous *stabbings*, common to this drug and Bry., are distinguished by those of Acon. being as if made with a *narrower instrument*, while those of Squill are broader. It is important to remember these differences when dealing with the pains of pleurisy. In lung affections the shootings or prickings are *fine* which are met in cases calling for Acon. as compared with those which call for Bry. A careful study of the symptoms of pneumonia and of these two drugs will show the place of Acon. is, and *only*, in the *early* stage of the attack, while that of Bry. may be in that which is next later than the Acon. stage. The pains of these two are aggravated by every motion; while many pains of Bell., especially those in the chest, are not aggravated by the movements of respiration [see Seneg.]. The *sticking* pains with Bell. are more *beneath the sternum*, or these may be as of the *cutting of knives*, or of a *sticking*, *pinching* character, and on *either side* of the *upper portion* of the sternum. The pains of Bell. are often of a *boring* character; those of Bry. are not, and this is but slightly represented in the pains of Acon. Bell. is more representative of *pressing pains* than either of the others. But its pains are chiefly *sticking*, or of a *tensive*, *drawing*, *tearing* character, and all are greatly aggravated by moving the *painful part*, and by *touch* or *pressure*, and, conversely, they are relieved by repose.

Then, in the elements of the febrile condition, where the liability of the novice to make a wrong selection is very considerable, and where, if the wrong drug is given, the embarrassment it will cause in the subsequent treatment is often great and sometimes the mistake is fatal, there should be great care in the examination of the symptoms of the case, and in discriminations of the differences which pertain to these and to the drug symptoms, which are otherwise very like in each.

One of the first elements likely to engage the attention of the prescriber in these cases is the pulse, and how very like it is in each in that which is likely first to arrest the attention of the prescriber. The three have *full*, *hard*, and *quick* pulse. This is sometimes intermittent with Acon. and Bry., with Bell not. Acon. has cold sensation in the blood-vessels. Neither of the others have this. It is not to be understood that Acon. is to be rejected in cases otherwise calling for it if this symptom be absent, but in cases where the other symptoms are so much like those of the two drugs as to leave doubt as to which should be selected this sensation of coldness in the veins, if present, will decide the choice for Acon.; so in similar cases if the pulse intermits. This will exclude Bell.; but as this fact is met in the record of both Acon. and Bry., the decision as between these must be governed by other symptoms. If the pulse be slow, and at the same time full, the case excludes both Acon. and Bry., but requires Bell. If there be a tense feeling of the pulse, this excludes Acon. It is found with both Bell. and Bry., and, as in other cases of similarity, the choice is decided by other symptoms. If there be ebullition of the circulation, Bry. will have the preference.

After the pulse, the other elements of the febrile paroxysm are likely next to engage attention. Important inflammatory affections which oftener call for one of these three remedies than for others, especially in their early treatment, are for the most part initiated with a chill, and this presents many differences in its concomitants which are important as differentiating elements in the selection of the specific remedy. Acon. in the initiation of sicknesses has severe chill in the *evening*, after *lying down*, with *hot cheeks* and *contracted pupils.* Chill from being *uncovered* or *touched.* During the heat chill occurs on the *slighest motion.* *With* the *chill* there is often *internal heat.* Shuddering runs *from the feet* to the chest.

With Bell. the evening chill *attacks the extremities*, especially *the arms*, with *heat of the head.* It has *internal chill* with *external burning heat.* Chill and heat *alternate.* Shudderings run *down* the back. This is the opposite of Acon., which rises from the feet upward.

Bry., chilliness, with *heat of head and face* [Bell., with cold extremities]; chills, with simultaneous heat; chills, with desire for *cold drinks*, and takes *much at a time.*

The heat produced by Acon. is *dry* and *burning*, proceeding, for the most part, *from the head and face*, with thirst for *cold drinks.* There is also great excitability; restlessness, with anxious tossings about; continued external heat, with disposition to *throw off the covering.* With the *burning heat*, there may be *concomitant cold shudderings.*

Bell. has *dry, burning heat*, with *sweat* only *on the head; internal heat*, with anxiety and restlessness; *heat* of the *forehead*, with *cold cheeks; internal* or *external* heat, or *both* at the *same*

*time; heat* of the *head,* with *red cheeks* and *delirium. Heat* is the *predominant* element of the paroxysm.

Bry., heat, *dry* and *burning,* mostly *internal;* blood seems to *burn in the veins* [Acon., it seems cold]; *heat in head in the forenoon,* feels as if it would *come out* at the *forehead; heat,* with desire to *uncover* [Acon.]; *heat,* with *thirst; great aggravation* of the sufferings *during the heat.*

Sweat from Acon. may be *over the whole body,* and of a *sour smell,* or it may be most on *covered parts;* or the *hot skin* is covered with a perspiration, which is also *hot.*

Bell. has *sweat* exclusively on *covered parts,* but it does *not smell sour;* or, it may have perspiration *with,* or immediately *after,* the heat, and *most on the face; empyreumatic*-smelling sweat, which *stains the linen;* sweat *in sleep,* day and night; sweat *ascends from the feet* to the head.

Bry., sweat *in the morning; cold* sweat *on the forehead* and whole head; in *brief spells,* and only *on single parts;* profuse *after 3 o'clock A. M.;* sweat profuse, *sour,* or *oily;* sweat easily produced *by exercise,* even in cold air; sweat over whole trunk and head, *except on affected parts; sweat relieves;* no thirst with the sweat.

Then these three remedies agree in the time of day in which their attacks, or the greater violence of these, are met—each prefers the evening and night. Acon. is at its worst at *night;* both Bell. and Bry. in the evening, though their attacks may be extended into the night. Acon. and Bry. are relieved while sitting, Bell. is not. Sufferings from Acon. are much relieved in the open air, those of Bell. and Bry. are but slightly so. Acon. is much better while sitting, Bry. less so, and Bell. not at all. The parts affected by these three remedies are sensitive to touch and motion—Bell. more to touch than the other two, and these more to motion than Bell. Indeed, some of the pains of Bell. are relieved by motion, especially those in the chest [see Seneg.]. Another peculiarity of Bell. is sensitiveness to touch; a slight, or even the slightest, touch is intolerable, while a firm grasp is much less hurtful [see Chin.]. The pains of Bell., often when they have reached their greatest intensity, suddenly cease, and they often suddenly disappear from one part to appear as suddenly in another. Neither Acon. nor Bry. are characterized by either. Bell. and Bry. develop sufferings more during sleep; Acon., both during and after sleep.

If we turn to the examination of the effects of these remedies on particular organs or functions, and begin with pains in the head, we shall find those of Acon. *increased* by *walking* in the

*open air;* by *motion, drinking, speaking,* and by a *current* of air; Bell., by *motion, walking, after walking* in the *open air*, when the *head* is *covered, stooping, laughing, stepping, going up stairs, external pressure, warmth of bed, lying down, moving the eyes, shaking the head, current of air;* Bry., first *opening of the eyes* in the *morning, moving the eyes, eating, stooping, rapid walking, lying* on the *back*.

The different kinds of pain in the head caused by Acon. are *bruised, fullness, heaviness* in the forehead, as if the *eyes would fall out*, or as if the brain *pressed out; tensive, constrictive, squeezed together*, or *contractive* pains. *Shootings, throbbing, burning*, as if the brain were *movable in the skull; one-sided, drawing, tearing, pulling, jerking, shooting.*

Bell. has *numbness* [see Plat.], *heaviness*, as if drunk; *pressing*, like a stone in the forehead [typhoid]; *tensive, bursting, swashing*, as if from water in the brain; *drawing, tearing, shooting, cutting, boring, throbbing.*

Bry. has *heaviness, fullness, pressure, squeezing, pressing asunder, tearing, shooting, throbbing,* and *burning.*

It will be seen that several of these pains are repeated in the record of each of the remedies. In prescribing for sicknesses where these are met, the differentiation is determined by the concomitants of the head symptoms, or by the more general and constitutional symptoms of the case. Those pains which are only found in the record of one are of first importance, and are often decisive of the choice.

Organs of optical sense should receive a twofold consideration when studying them in their relations to diseases and curatives. They have the same susceptibility to impressions from morbid causes as other material tissues of the organism, and are, like other organs, subject to pains, inflammations, and changes of tissue, and also to modifications of the functions of their special sense. Thus in the eye we have from Acon. *pressure, burning, inflammation* (this is common to the three remedies. For differentiation see concomitants), *as if* the eyes were *swollen, swelling of inflamed eyes, red veins* in inflamed eyes, *dizziness* and *heaviness, painful, tense, hard swelling of eyelids.*

*Photophobia, desire* for *strong light.* [See Bell.] *Black spots* and *cloud* before eyes, *clouded vision*, great *keenness* of *vision.*

With Bell., pains *in the orbits*, as if the *eyes would be torn out* or *pressed into* the orbit. *Pressure*, as if from *hand, pressure* in the *eyeball. Tearing, drawing, smarting, shooting, burning* pains. *Dryness, lachrymation, injected conjunctiva.* Inflammation of eyes and also of lids, with swelling and throbbing pains. [It may be remarked, in the general, that in inflammations of the eyes and their appendages, Acon. and Bell. affect the more superficial tissues, and Bry. those deeper seated. Bell. affects more the brow and the orbit than either of its near relatives. The same may be said of the lids, except that Bry. has more affinity for the upper lid than either of the other two. Bell. affects the *margins* of the lids more than Acon. or Bry. Acon. and Bell. are more related to affections of the *inner surface* of the lids than Bry. Bell. and Bry. are more in relation to affections of the angles of the lids than Acon.; and that Bell. is especially called for in affections of the *inner*, and Bry. in those of the *outer*.]

Inflammation of the *carunculæ*, with *swelling* and *suppuration; burning pressure, softening* of the *sclerotica, thickening, specks* and *ulcers* on the *cornea; bleeding* and *euchymosis* of the *conjunctiva, yellow* conjunctiva, *everted lids, heaviness* of *lids, cramps* of *lids, falling* of *upper lid*, as if paralyzed; *pupils* much *contracted* or *dilated; desires to look at strong light*, or *strong photophobia; dull vision*, as if *through a cloud; black before the eyes; unable to read, can only see a white border* of the letters; the letters are *gold* or *blue* colored while reading; *bright red* corona *around the light; flames, silver cloud*, and *sparks* before the eyes; *white stars* on the ceiling of the room; *double vision;* objects appear *disordered* or *red; amaurosis, strabismus; weakness* of vision.

It is easy in the above group of most important eye symptoms to see its broad relations to optical diseases. There is, perhaps, no more important remedy available in clinical dealings with these, but this group, properly studied, will show that its use is far from being limited to local eye affections or functional defects of this organ.

Bry. has as if the eyes were *pressed out* from the head; pressure as if *from sand; eye-balls painful to pressure; shootings; burning* in *the edge* of the lids; *throbbing; inflammation*, especially of the deeper tissues of the eye. [The differentiation of these remedies in their relation to particular cases of ophthalmia must be made by reference to their concomitant symptoms as found in the materia medica. These inflammations are not repeated identities, as they are met in successive cases, and it does not fulfill our duty when before a case to be prescribed for to say inflammation, and therefore one of these remedies must be given. This is far away from duty well performed. It may be the case requires neither, and if either, it is certainly not a matter of indifference which. There should be the sharpest individualization of the case, and then it should be put on trial before these groups and *their concomitants* before either is given, and then, if either, it is to be that which is most like these, and for this reason and no other.] *Fullness* as if the eyes were pressed out; *shootings; jerkings; swollen lids,* especially the *upper; lachrymation; agglutination* of the lids at night; *photophobia; illusions in bright colors; flickering; rainbow* colors; objects appear *covered* with the *colors of the prism; letters run together;* dim vision; bluish haze. It may be well to remember that nearly all the pains of eye affections, as well as many of those of the head, which call for Bry., are aggravated by *moving the eyes.*

In action on the ear and its special function, it will be found most symptoms, and those of most importance, are in the record of Bell. This remedy belongs to ear affections which are more of a simple or local character, while those of Bry. are oftener complicated with other and more important elements of sickness; as, for instance, its deafness and intolerance of sound are found in typhoid complications and in inflammatory conditions of the brain.

Acon. has *tearing* pain or *tickling* as if from the *crawling* of a worm; *pressing pain* behind the left ear; *burning* in left; *ringing, roaring, stopped,* or as if a *skin* were *drawn before the ears; increased sensibility* of hearing; every noise is insupportable.

Bell. has *squeezing* and *stiffness* in inner ear, with shocks; *pressure,* internal and external; *tearing pains,* as if the ear would be *torn out;* also in temples, with *pressing inward* and alternating with similar pains in the orbits; *stitches; pressure* in *mastoid* process, or *cutting blows* from without inward; *boring* near the ear; *pressing, tearing* behind the ear; *drawing* from the ear to the neck; *pinching* in the ear, *with hiccough; purulent discharge; inflammation; ringing; noise* like a *trumpet* and *drumming; rushing, roaring, buzzing, hammering, fluttering wind passes from the ear; deafness, hard-hearing* from *cold;* increased *sensitiveness* of *hearing* and *aversion to noise; stitch* in the *parotid gland* daily at the same hour; *swelling* of the *parotid,* also inflammatory.

Bry. has *pressure* in the *external* ear; *contracting* pain with *deafness; pinching* near the *ear* and in the *joint of the jaw; stitches, burning, hard swelling* behind the ear; *chapping, moist, yellow-crusted* eruption before the ear; *bleeding* from the ear; *ulcerated external* ear; *deafness,* as if *stopped. Rushing, buzzing, ringing, noises* are *unendurable.*

The organ of the sense of smelling is not so affected by these remedies as to develop many or so important symptoms as are found in the record of their actions on the other organs of special sense, yet there are some which are significant of relationship to sick conditions. Acon. is related to *hæmorrhages* from the nose in *plethoric subjects.* Its action on the olfactory organ, producing extreme *sensitiveness to odors,* may be accepted as a reason for selecting this remedy and excluding others which, together with Acon., have symptoms similar to those of the case in hand. *Violent sneezing* which causes *pain in the abdomen* may warrant its selection for influenzas so characterized.

Bell. has *pain* as if *bruised* when *touched; drawing* in the *left half; pressure* in the *bones; crawling* in the *end* of the nose, or *stitch,* from the evening through the night; the *end* of the nose is very *cold,* or *red* with *burning* and *sweat* [typhoid]; *painful ulceration of the nostrils; red nodules* on the *root of the nose,* with pain, as from ulceration, when touched; *scabby pustules* on the nose; *bleeding* from the *nose,* especially *mornings* or *nights; hæmorrhage* from the *nose* and *mouth;* great *sensibility* of the sense of *smelling;* the odor of *soot* and *tobacco* is *unendurable; smell from the nose* like *spoiled eggs; diminished sense* of *smelling; frequent sneezing;* great *dryness* of the nose; *alternating obstruction* and *flow of water; coryza* with *cough,* and with *smell of herrings* before the nose; *fluent coryza* from only *one side.*

Bry. has *swelling* of the nose, with severe *ulcerative pain* when *touched; twitching* in the nose, with old, *dry coryza; inflamed* and *ulcerated nostril,* with *smarting;* frequent and violent *bleeding* from the nose, mornings,* with yawning; violent and frequent *coryza,* for the most part *fluent,* with *stoppage* of nose and *chill,* with *much sneezing,* with *shooting* in the *head* or *pain* in the *forehead,* as if all would burst out there, especially while *stooping;* long-continued, *dry coryza; hard plugs of mucus* in the nose.

The differences of the effects of these three drugs on tissues of the nose or of their modifications of its special sense are so plain as to need no reference to them. It is enough to read the record. Many of those of Bell. are not found in the records of either of the others. The same may be said of those of Bry. The hæmorrhages, coryzas, and acute or diminished sense of smell are to be taken, with their concomitants, in deciding whether either of these or some other remedy is to be given for the cure.

---

* In typhoid fever, hæmorrhage from the nose *after four o'clock in the morning* calls for *Rhus. tox.* Not unfrequently where this symptom is met a single dose of a rightly selected potence of this drug will cut short the typhoid process and the convalescence will be surprisingly prompt, brief, and perfect. We have repeatedly seen this result in most unpromising cases.

[TO BE CONTINUED.]

